# Reversibility of Hypercapnia after an Acute Exacerbation of COPD

**DOI:** 10.1159/000524845

**Published:** 2022-06-03

**Authors:** Jens Bräunlich, Kristin Turba, Hubert Wirtz

**Affiliations:** ^a^Department of Respiratory Medicine, Emden Hospital, Emden, Germany; ^b^Department of Respiratory Medicine, University of Leipzig, Leipzig, Germany

**Keywords:** Chronic obstructive pulmonary disease, Exacerbations, Respiratory muscles, Noninvasive ventilation

## Abstract

**Background:**

After an episode of hypercapnic AECOPD, some patients show reversible, prolonged or persistent hypercapnic respiratory failure. However, at the time of patient discharge, it is uncertain whether patients will remain hypercapnic or may return to a physiologic gas status.

**Methods:**

Data were retrospectively collected from COPD patients with an acute hypercapnic exacerbation (AECOPD). Out of 143 total COPD inpatients, complete data set was available for 82 patients in stable condition. According to the first available capillary or arterial pCO<sub>2</sub>, patients were divided into those with persistent hypercapnia (PHG) and those with reversible hypercapnia.

**Results:**

In this study, 51% of patients with acute hypercapnic AECOPD and follow-up (FUP) visits developed normocapnia after a time period of several weeks. These patients were characterized by lower carbon dioxide partial pressure (PaCO<sub>2</sub>), HCO<sub>3</sub>^−^, and BE levels prior to the AECOPD event, at discharge and at FUP. pH was higher at discharge and FUP in this group. Greater disease severity and lower forced vital capacity were prominent in patients with PHG. Binary logistic regression revealed GOLD D and higher PaCO<sub>2</sub> at discharge as predicting factors for PHG.

**Conclusions:**

A large percentage of patients has prolonged hypercapnia following acute hypercapnic COPD exacerbation. The risk profile of patients with irreversible hypercapnia should be carefully evaluated following AECOPD in order to choose selected patients for home-noninvasive ventilation.

## Introduction

Patients with chronic obstructive pulmonary disease (COPD) suffer from worsening of clinical parameters during an acute exacerbation (AECOPD). These life-threatening events are often associated with respiratory muscle weakness and increasing hypercapnia. Noninvasive ventilation (NIV) is an established method to treat respiratory failure in a multiplicity of diseases. NIV is recommended in acute hypercapnic and acidotic respiratory failure during AECOPD [1]. NIV application in chronic hypercapnic respiratory failure due to COPD was investigated extensively in recent years with documented clinical benefits [2, 3]. Recommendations about the long-term use in stable hypercapnic COPD have been published. According to this recommendation, hypercapnia is the prominent parameter to initiate home-NIV [4].

Following an episode of hypercapnic AECOPD, some patients show reversible, prolonged or persistent hypercapnic respiratory failure. However, at the time of patient discharge, it is unclear whether the patient will remain hypercapnic or may return to a physiologic gas status. Selection of patients for permanent home NIV treatment at this time is an unsolved problem. The study by Struik et al. [5] revealed this problem. Little benefit and no difference in terms of improvement in hypercapnia was observed in patients with early NIV following a hypercapnic AECOPD event when compared to those without this treatment. There is an ongoing discussion on patient selection for chronic NIV treatment following a hypercapnic episode and the optimal time point to initialize chronic NIV treatment.

Reversible hypercapnia (RHG) has been subjected to several clinical studies, with as many as 40% of all patients exhibiting RHG. Factors predicting the development of RHG were higher forced expiratory volume in 1 s (FEV1) or absent Cor pulmonale [6, 7, 8].

Clinical criteria and individual characteristics useful to predict permanent hypercapnia may be helpful to clinicians. This study tries to express that lots of COPD patients show RHG after an acute exacerbation. According to previous studies, we hypothesize that persistent and reversible hypercapnic patients show differences in paO_2_, carbon dioxide partial pressure (PaCO_2_), lung function, and other values.

## Methods

**Fig. 1:**
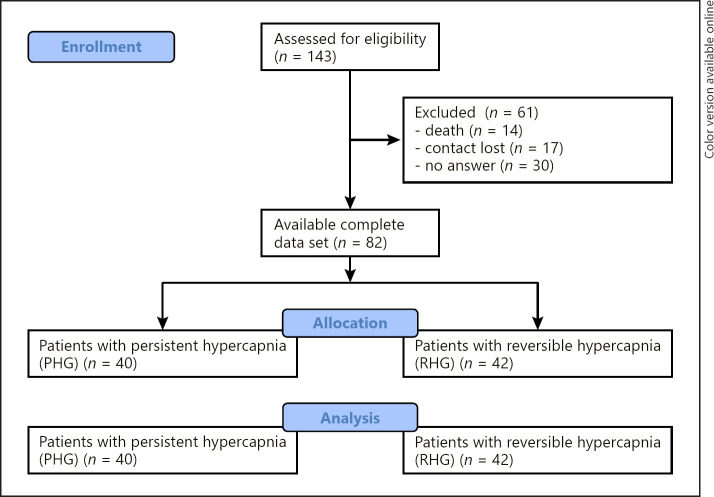
CONSORT flowchart of the study.

We collected data retrospectively from COPD patients with an acute hypercapnic exacerbation (AECOPD), treated as inpatients at the University Hospital of Leipzig between August 2010 and March 2018. Out of 143 total COPD inpatients, complete data set was available for 82 patients in stable condition and could thus be included for further analysis. Sixty-one patients had to be excluded due to missing data (Fig. 1). According to the first available capillary or arterial pCO_2_, patients were divided into those with persistent hypercapnia (PHG) and those with RHG. The majority of data was carried out in ambient air; however, some (very ill) patients received 0–4 L of oxygen. For data collection, we used clinical records including laboratory values and results from lung function testing. Hypercapnia was defined as an arterial or capillary PaCO_2_ above 45 mm Hg, and COPD severity was classified by GOLD. AECOPD was diagnosed by using the Anthonisen criteria. Other reasons for respiratory insufficiency were excluded by radiographic, laboratory, cardiological, and other necessary examinations. In most cases, a specific cause of exacerbation was not identified. We collected patient demographics, clinical, and functional data and used MS Excel to organize it.

### Statistical Analyses

All statistical tests were performed using IBM SPSS Statistics for Windows, Version 24.0. Descriptive statistics are presented as mean ± SD or median and interquartile range. Normality of distribution was estimated visually from boxplots and histograms. We compared baseline characteristics of our sample (82 follow-up (FUP) patients, divided into PHG or RHG) by using the independent samples *t* test in case of metric variables or the Fisher's exact test in case of categorical data. All tests were performed two-tailed and a *p* value <0.05 was taken as statistically significant.

In a second analysis, we performed a binary logistic regression to assess the prognostic value of the patient characteristics (as independent variables, i.e., predictors) on the chance of PHG after AECOPD (as a dependent variable). Independent variables associated with PHG in the univariate analysis, as well as age, gender, and BMI, were entered in the final binary logistic regression model.

## Results

As presented in Figure 1, 143 AECOPD patients with acute hypercapnic respiratory failure were enrolled, of whom 79% (113/142) were discharged with hypercapnia and 21% (29/142) with normocapnia. One patient had to be excluded due to missing data.

In total, we lost 61 patients due to missing FUP data, whereas 82 patients could be included in the primary analysis (including the 1 patient who did not have any values at discharge). Despite ABG by the time of discharge, we included all patients in further statistical analyses (including patients with normocapnia at discharge). The mean time to FUP was 147.5 days (CI: 103.6–191.5) after discharge. At that point, 40 patients (49%) were identified to have PHG and 42 patients (51%) reverted to normocapnia, which was depicted as RHG. Seventy-one of these 82 FUP patients (88%) were hypercapnic on discharge. In this subgroup 39% reverted to normocapnia at FUP. Nineteen of these hypercapnic patients were treated with NIV after AECOPD; 16 remained hypercapnic (hypercapnia despite of NIV and therefore classified as persistent hypercapnic) at FUP and 3 reverted to normocapnia. Twenty-eight patients were normocapnic before AECOPD, and 9 of these patients turned to PHG at FUP.

As stated in Table 1, sex distribution did not differ significantly between groups (54% male and 50% male). The mean age was 69.4 years in the group with RHG and 67.1 years in the group with PHG, indicating no significant difference. BMI, cigarette pack years, or smoking status were not different in the two groups.

GOLD D was significantly more prevalent in the PHG (*p* = 0.048). In addition, the following values retrospectively evaluated from time points before AECOPD were significantly higher in PHG than in RHG: PaCO_2_ (*p* = 0.023), HCO_3_^−^, and BE (*p* = 0.002 and *p* = 0.003). In contrast, paO_2_ and pH levels before AECOPD were similar in both groups. Patients in the PHG also had higher PaCO_2_ (*p* = 0.021) and HCO_3_^−^ levels (*p* = 0.022) at the time of admission, while paO_2_, pH, and BE levels were similar.

The two groups differed at the time of discharge: PaCO_2_ level was significantly higher (*p* < 0.001) in the PHG group, as well as HCO_3_^−^ and BE levels (*p* = 0.001 and *p* = 0.007), while pH level was significantly lower (*p* = 0.030). PaO_2_ level yielded no statistically significant difference.

As we had expected, significant differences existed between groups at FUP: PaCO_2_, HCO_3_^−^, and BE levels were significantly higher in the persisted hypercapnia group, while pH levels were lower (*p* each <0.001). PaO_2_ was similar in both groups.

Forced vital capacity (FVC) was lower even prior to AECOPD in PHG (mean time: 327.1 days [CI: 218.6–435.6]) and also months after AECOPD: mean 159.9 days following discharge (CI: 101.6–218.1; *p* = 0.048). FEV1 and the FEV1/FVC ratio were not different. No difference was observed between the two groups regarding the frequency of pulmonary hypertension, the prevalence of right heart decompensation, and medication.

### Binary Logistic Regression Model

The binary regression was performed to identify factors associated with the likelihood of PHG after AECOPD (Table 2). For the logistic regression model, the binary dependent variable (PHG vs. RHG) was coded as positive (PHG) or negative (RHG) towards potential risk factors as independent variables, i.e., predictors: sex, age, BMI, pack years, smoking habits, GOLD D, FVC, FEV1/FVC, PaCO_2_, and HCO_3_^−^ by the time of discharge. Due to missing data, the analysis includes 63 patients.

The model was able to correctly classify 75.0% of patients with PHG and 80.6% of patients with RHG, resulting in an overall success rate of 77.8%. Nagelkerke's *R*^2^ was 0.455, which is considered a strong effect. The full model containing all predictors was statistically significant (χ^2^ [10] = 26.28, *p* = 0.003, *n* = 63), as well as the variables PaCO_2_ and GOLD D (*p* = 0.012 and *p* = 0.028).

Based on odds ratio, the model indicates that patients with higher PaCO_2_-level by the time of discharge and patients in GOLD D were 1.2 and 8.2 more likely to remain hypercapnic compared to RHG. All other variables were not significant as predictors of PHG.

## Discussion

In this study, 51% of patients with acute hypercapnic AECOPD and FUP visits developed normocapnia after a time period of several weeks. These patients were characterized by lower PaCO_2_, HCO_3_^−^, and BE levels prior to the AECOPD event, at discharge and at FUP. pH was also higher at discharge and FUP in this group. Greater disease severity and lower FVC were prominent in patients with PHG. The binary logistic regression model revealed GOLD D and higher PaCO_2_ at discharge as predicting factors for PHG.

McNally et al. [7] described RHG in 40% of patients with hypercapnic AECOPD. These patients had higher arterial oxygen tension at admission, more normal pulmonary function, lower BMI, and less pulmonary hypertension. Our study did not identify BMI, pulmonary hypertension or PaO_2_ as characteristic factors for RHG. But we found a lower HCO_3_^−^ and BE more prevalent in reversible hypercapnic patients. In Costello's study, patients with Cor pulmonale placed on LTOT, lower PaO_2_ at admission as well as discharge, and a higher PaCO_2_ at discharge had a higher risk to develop PHG [6]. These patients, also characterized by reduced lung function, exhibited inferior survival and a higher rate of readmission. However, patients with RHG did not have an increased risk of death compared to eucapnic AECOPD patients. In our study, there was no significant difference in FEV1 between reversible and persistent hypercapnic patients. But we identified FVC as being significantly lower in persistent hypercapnic patients. McNally et al. [7] reported a comparable 40% hypercapnia reduction (defining normocapnia at 45 mm Hg or below). Costello et al. [6], in contrast, defined normocapnia as a value of 50 mm Hg and below. It is therefore likely that the number of normocapnic patients would even be in excess of the reported 54% when using 45 mm Hg as a threshold [6, 7]. A very recent study addresses the problem of clinical and laboratory findings in stable hypercapnic patients. A significant association with the presence of hypercapnia was found for a higher BMI, a lower FVC and higher bicarbonate HCO_3_^−^ levels [9]. The authors found 25% of GOLD 3 out of 4 patients were hypercapnic in this outpatient evaluation. These findings in FVC, GOLD stage, and HCO_3_ levels are in line with our study results.

Our results complement and confirm these previous studies, but add new and important aspects. In our study, we also found reduced lung function and higher PaCO_2_/bicarbonate at discharge in ongoing hypercapnic patients. Our study expands former study results and adds a higher GOLD stage (D) and hypercapnia before an AECOPD event to the risk factors for developing stable hypercapnia. This additional information allows us to identify patients with the risk of ongoing hypercapnia more exactly and earlier. An immediately initiation of a NIV in these patient group could prevent next exacerbation.

The central focus following an AECOPD episode is the prevention of another. Repetitive AECOPD is associated with a bad prognosis and a loss of lung function [10]. Among other lines of treatment, it has been reported that the initiation of NIV at home is effective [4]: a randomized controlled trial by Murphy et al. [2] documented improvements in terms of the combined endpoint of readmission/death after including patients for more than 2 weeks after AECOPD. Köhnlein et al. [3] conducted a trial that revealed significant differences in mortality rates in stable hypercapnic COPD patients receiving NIV at least 4 weeks following an AECOPD episode. This is in contrast to the study by Struik et al. [5]. Patients in this study with prolonged hypercapnia after hypercapnic AECOPD were rapidly (>48 h after respiratory support) randomized to NIV or standard treatment. At 1 year following treatment, there was no improvement in the time to readmission or death between the two treatment groups [5].

Although these important studies are not entirely comparable, they illustrate the problem of patient selection and the correct time-point for initiating continuing NIV. However, it is also a definite finding of this and similar studies (see above) that a considerable group of patients improves and regains their functional ventilatory status following AECOPD.

Our study supports the idea that the time to the last hypercapnic exacerbation should be long enough to document a stable hypercapnia. In our study, the median FUP time was 148 days with a threshold of 45 mm Hg or below, defining as normocapnia. However, our study does not aide in clarifying the exact time for defining permanent hypercapnia but does expand our knowledge with regard to the question of how many patients will return to a normocapnic state and what characterizes these patients. As far as we know, this is the first study focusing on different times for ABG data, i.e., time before and weeks after the index hospitalization.

Characterization of patients with imminent or already present PHG prior to and also following AECOPD is an important aspect of COPD specific care. In the wake of an AECOPD episode, patients should be carefully monitored for weeks following the event. The characterization of and thus the possibility of concentrating on those patients mostly endangered by permanent respiratory insufficiency will save resources and provide a treatable trait for highly selected COPD patients in terms of hospital readmission and death.

Our study has several limitations. First, the study design was retrospective. This leads to several problems during data collection. For example, data about oxygen supplementation were sometimes missing in outpatients. Second, we had some missing FUP data, which leads to a possible bias. Third, the panel of parameters was limited by the availability of the data. Fourth, the long time-span between the first occasion and discharge and the last occasion was the consequence of the frequency of ABG evaluation. In some patients, the adherence to the pulmonologist was poor or the pulmonologist/practitioner only evaluated lung function data without routine ABG measurements. This fact mainly explains the long time between the ABG measurements and reflects the daily practical problems in this patient group. We looked for promptly available ABG data before and after the index hospitalization. In addition, we contacted the patients, the practitioner, or pulmonologist for further data to minimize the time span. Despite this careful data collection, we cannot close out events between the ABG measurements. This weakens the findings of our study.

## Conclusions

We observed a large percentage of patients with only transient hypercapnia following acute hypercapnic COPD exacerbation. The risk profile of patients with irreversible hypercapnia is described in this study and should be carefully evaluated following AECOPD in order to choose selected patients for home-NIV that will benefit from this treatment.

## Statement of Ethics

The study was approved by the Ethics Committee of the University of Leipzig in 2014 (324/16-ek). No written, informed consent was necessary because data was collected retrospectively and kept anonymous.

## Conflict of Interest Statement

All the authors declare no conflicting interests regarding the study. Hubert Wirtz is an Editorial Board Member of *Respiration*.

## Funding Sources

This study has no funding sources.

## Author Contributions

All the authors Jens Bräunlich, Kristin Turba, and Hubert Wirtz fulfill ICMJE criteria for authorship. Jens Bräunlich and Kristin Turba shared first co-authorship.

## Data Availability Statement

All data generated or analyzed during this study are included in this article. Further inquiries can be directed to the corresponding author.

## Figures and Tables

**Table 1:** Baseline characteristics of the study cohort

	Total (*n* = 82)	Patients with RHG (*n* = 42)	Patients with PHG (*n* = 40)	*p* value
Age, years^1^	68.3 (10.1)	69.4 (9.4)	67.1 (10.8)	0.312
Gender (M), *n* (%)	43 (52)	23 (54)	20 (50)	0.825*
BMI, kg/m^2^ ^1^	*n* = 80	*n* = 42	*n* = 38	0.426
	25.5 (5.7)	25.1 (5.8)	26.1 (5.6)	
Pack years^2^	*n* = 74	*n* = 38	*n* = 36	0.943
	40 (25–50)	34 (20–46)	40 (30–50)	
Current smoker, *n* (%)	*n* = 79	*n* = 40	*n* = 39	0.586*
	17 (21)	10 (25)	7 (18)	
GOLD D, *n* (%)	*n* = 80	*n* = 40	*n* = 40	0.048*
	64 (80)	28 (70)	36 (90)	
Right heart decompensation, *n* (%)^a^	32 (39)	19 (45)	13 (33)	0.265*
LTOT before, *n* (%)	*n* = 72	*n* = 34	*n* = 38	0.058*
	30 (41)	10 (29)	20 (53)	
NIV during event, *n* (%)	42 (51)	20 (48)	22 (55)	0.517*
PaCO_2_ before event, mm Hg^1^	*n* = 60	*n* = 29	*n* = 31	**0.023**
	48.2 (11.5)	44.8 (7.1)	51.4 (13.8)	
PaO_2_ before event, mm Hg^2^	73.0 (38.3)	71.6 (29.5)	74.2 (45.2)	0.792
pH before event^1^	7.42 (0.05)	7.43 (0.05)	7.41 (0.06)	0.274
HCO_3_^−^ before event, mmol/L^1^	31.0 (4.8)	29.0 (3.6)	33.0 (5.0)	**0.002**
BE before event, mmol/L^1^	6.2 (4.2)	4.5 (3.2)	7.8 (4.3)	**0.003**
PaCO2 admission, mm Hg^2^	55.5 (49.0–68.8)	53.0 (47.9–61.1)	59.2 (50.4–76.5)	**0.021**
PaO_2_ admission, mm Hg^2^	66.0 (54.6–83.7)	70.5 (55.6–82.5)	62.1 (52.7–85.2)	0.265
pH admission^1^	7.35 (0.09)	7.36 (0.09)	7.34 (0.10)	0.185
HCO_3_^−^ admission, mmol/L^1^	32.6 (5.0)	31.3 (4.3)	33.9 (5.4)	**0.022**
BE admission, mmol/L^1^	6.5 (4.8)	5.8 (4.7)	7.3 (5.0)	0.209
PaCO_2_ discharge, mm Hg^1^	51.4 (7.7)	48.0 (6.1)	54.9 (7.6)	**<0.001**
PaO2 discharge, mm Hg^1^	62.3 (20.0)	66.5 (20.0)	58.0 (19.2)	0.053
pH discharge^1^	7.43 (0.04)	7.44 (0.05)	7.42 (0.04)	**0.030**
HCO_3_^−^ discharge, mmol/L^1^	33.5 (4.0)	32.1 (3.7)	34.9 (3.8)	**0.001**
BE discharge, mmol/L^1^	8.7 (4.0)	7.4 (4.1)	9.9 (3.6)	**0.007**
PaCO_2_ FUP, mm Hg^2^	44.9 (40.8–53.1)	40.9 (39.0–43.1)	53.5 (48.1–58.6)	**<0.001**
PaO_2_ FUP, mm Hg^1^	71.6 (48.6)	70.7 (17.3)	72.5 (67.7)	0.872
pH FUP^1^	7.42 (0.04)	7.44 (0.04)	7.40 (0.04)	**<0.001**
HCO_3_^−^ FUP, mmol/L^1^	29.1 (4.3)	27.0 (3.4)	32.3 (3.4)	**<0.001**
BE FUP, mmol/L^1^	4.3 (3.8)	3.0 (3.5)	6.7 (3.0)	**<0.001**
FEV1 (% predicted)^1^,^b^	*n* = 72	*n* = 35	*n* = 37	0.189
	35.4 (16.1)	38.0 (16.5)	33.0 (15.5)	
FVC (% predicted)^1^,^b^	49.7 (16.8)	53.7 (16.7)	45.9 (16.2)	**0.048**
FEV1/FVC, %^1^,^b^	52.8 (15.2)	52.7 (14.9)	52.9 (15.6)	0.976
RV (% predicted)	220.1 (72.4)	208.4 (70.9)	231.3 (73.2)	0.234
RV%TLC (% predicted)	157.6 (49.0)	148.4 (50.8)	166.4 (46.4)	0.169
Pulmonary hypertension, *n* (%)^c^	19 (23)	8 (19)	11 (28)	0.437*
Medication, long-time therapy, *n* (%)				
Inhaled corticosteroids	59 (72)	30 (71)	29 (73)	0.915*
Oral corticosteroids	66 (80)	31 (74)	35 (87)	0.165*
LABA	75 (91)	38 (90)	37 (92)	0.745*
LAMA	74 (90)	36 (86)	38 (95)	0.265
Theophylline	8 (10)	4 (10)	4 (10)	0.942*
Antidepressants/antipsychotics	20 (24)	8 (19)	12 (30)	0.308*
Beta blockers	39 (48)	16 (38)	23 (58)	0.121 *

LTOT, long-term oxygen therapy; NIV, noninvasive ventilation; FEV1, forced expiratory volume in 1 s; FVC, forced vital capacity; RV, residual volume; TLC, total lung capacity; LABA, long-acting beta-agonist; LAMA, long-acting muscarinic antagonist. Data are presented as

1 ean (SD) or

2 edian (interquartile range). Divergences in “n” compared to total patients due to missing values. The values in bold are considered as statistically significant (*p* < 0.05).

* Fisher's exact test; rest: *t* test independent samples.

a Right heart decompensation signs such as edema.

b In stable conditions before or after event (mean: 327.1 days before AECOPD or 159.9 days after discharge).

c Echocardiographic mean time to FUP: 147.5 days after discharge.

**Table 2:** Prognostic value of PHG after AECOPD

	*B* (SE)	95% CI of *B*	*p* value	Odds ratio = Exp(B)
Gender (M)	0.250 (0.695)	0.329–5.017	0.719	1.285
Age, years	−0.017 (0.040)	0.909–1.063	0.672	0.983
BMI, kg/m^2^	−0.019 (0.072)	0.852–1.130	0.793	0.981
Pack years	−0.005 (0.015)	0.967–1.024	0.727	0.995
Current smoker	−1.416 (1.040)	0.032–1.863	0.173	0.243
GOLD D	2.107 (0.127)	1.253–53.934	**0.028**	8.221
FVC (% predicted)^a^	0.015 (0.024)	0.968–1.064	0.541	1.015
FEV1/FVC,^a^ %	0.039 (0.028)	0.985–1.098	0.160	1.040
PaCO_2_ discharge, mm Hg	0.209 (0.083)	1.048–1.450	**0.012**	1.233
HCO_3_^−^ discharge, mmol/L	0.067 (0.127)	0.834–1.371	0.595	1.070
Constant	−15.810 (5.746)		**0.006**	0.000
*R*^2^ (Nagelkerke)	0.455			
*n*	63			

The binary regression model was performed to predict the probability of PHG after AECOPD. The values in bold are considered as statistically significant (*p* < 0.05). FEV1, forced expiratory volume in 1 s; FVC, forced vital capacity.

a In stable conditions before or after event (mean: 327.1 days before AECOPD or 159.9 days after discharge).

## References

[1] Rochwerg B, Brochard L, Elliott MW, Hess D, Hill NS, Nava S (2017). Official ERS/ATS clinical practice guidelines: noninvasive ventilation for acute respiratory failure. Eur Respir J.

[2] Murphy PB, Rehal S, Arbane G, Bourke S, Calverley PMA, Crook AM (2017). Effect of home noninvasive ventilation with oxygen therapy vs. oxygen therapy alone on hospital readmission or death after an acute COPD exacerbation: a randomized clinical trial. JAMA.

[3] Köhnlein T, Windisch W, Köhler D, Drabik A, Geiseler J, Hartl S (2014). Non-invasive positive pressure ventilation for the treatment of severe stable chronic obstructive pulmonary disease: a prospective, multicentre, randomised, controlled clinical trial. Lancet Respir Med.

[4] Ergan B, Oczkowski S, Rochwerg B, Carlucci A, Chatwin M, Clini E (2019). European Respiratory Society guidelines on long-term home non-invasive ventilation for management of COPD. Eur Respir J.

[5] Struik FM, Sprooten RT, Kerstjens HA, Bladder G, Zijnen M, Asin J (2014). Nocturnal non-invasive ventilation in COPD patients with prolonged hypercapnia after ventilatory support for acute respiratory failure: a randomised, controlled, parallel-group study. Thorax.

[6] Costello R, Deegan P, Fitzpatrick M, McNicholas WT (1997). Reversible hypercapnia in chronic obstructive pulmonary disease: a distinct pattern of respiratory failure with a favorable prognosis. Am J Med.

[7] McNally E, Fitzpatrick M, Bourke S, Costello R, McNicholas WT (1993). Reversible hypercapnia in acute exacerbations of chronic obstructive pulmonary disease (COPD). Eur Respir J.

[8] Saryal S, Celik G, Karabiyikoğlu G (1999). Distinctive features and long-term survival of reversible and chronic hypercapnic patients with COPD. Monaldi Arch Chest Dis.

[9] Dreher M, Neuzeret PC, Windisch W, Martens D, Hoheisel G, Gröschel A (2019). Prevalence of chronic hypercapnia in severe chronic obstructive pulmonary disease: data from the HOmeVent Registry. Int J Chronic Obstruct Pulmon Dis.

[10] Osadnik CR, Tee VS, Carson-Chahhoud KV, Picot J, Wedzicha JA, Smith BJ (2017). Non-invasive ventilation for the management of acute hypercapnic respiratory failure due to exacerbation of chronic obstructive pulmonary disease. Cochrane Database Syst Rev.

